# 1310. Prediction of Surgical Site Infection Following Ankle Fracture: A Machine Learning Approach

**DOI:** 10.1093/ofid/ofad500.1149

**Published:** 2023-11-27

**Authors:** Nour Nassour, Alexander M Tatara, Sumner V Jones, Hadley Leatherman, William DiGiovanni, Soheil Ashkani-Esfahani, Sandra B Nelson

**Affiliations:** Massachusetts General Hospital, Boston, Massachusetts; Massachusetts General Hospital, Boston, Massachusetts; MGH, Cleveland, Ohio; Massachusetts General Hospital, Boston, Massachusetts; n/a, Stonington, Connecticut; Massachusetts General Hospital, Boston, Massachusetts; Massachusetts General Hospital, Boston, Massachusetts

## Abstract

**Background:**

Surgical site infections (SSI) are a common complication in foot and ankle surgery. In the US, ankle fracture incidence is 4.22/10,000 person-years. The development of a predictive model for SSI in foot and ankle surgery may provide insights that would enable the prevention of wound complications to reduce their burden. In this study, we analyzed a patient data set to compare 5 different machine learning models and determine which is most effective in determining the risk for surgical site infection after ankle fracture.

**Methods:**

We included 935 ankle fracture patients, of whom 114 patients developed SSI in the case group. We used the risk factors for SSI obtained from univariate analysis to develop 5 sets of models using the following machine learning (ML) methods: Decision Tree (DC), Random Forest (RF), Neural Network (NN), Gradient Boosting (GB), and Adaboost.

**Results:**

In univariate analysis, older mean age (58.1 y.o. vs 49.3 y.o, p< 0.001), higher mean American Society of Anesthesia score (2.1 vs 1.9, p< 0.001), and longer hospital stay relating to the ankle fracture (6.3 days vs 4.1 days, p< 0.001) were correlated with SSI risk when compared to non-infected patients. We also demonstrated that factors such as the presence of an external fixation prior to the ORIF (OR=2.91, p< 0.001), diabetes (OR=2.00, p=0.03), oral steroid use (OR=3.45, p< 0.001), multiple fractures (OR=2.29, p=0.003), liver disease (OR=2.76, p=0.02), hypertension (OR=2.05, p< 0.001), illicit drug use (OR=2.31, p=0.01), smoking (OR=1.71, p=0.02), open wounds (OR=3.32, p< 0.001), and CCI (OR=1.73, p=0.04) were also correlated with a higher incidence of SSI as compared to our control group. Our machine learning models performed well, with AUCs ranging from 0.74 to 0.86. GB had the highest AUC (0.86) and other favorable metrics including an accuracy of 0.88, F1-score of 0.89, precision of 0.91, sensitivity of 0.88, and specificity of 0.77 (table 1).

**Table 1.**
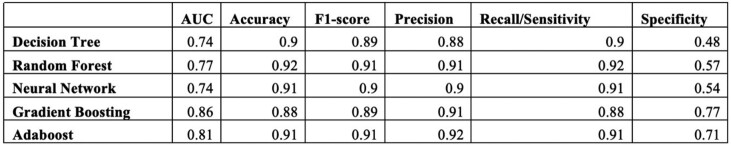
Performance Metrics of the Machine Learning Prediction Model for SSI following Ankle Fracture

**Conclusion:**

Early prediction of SSI risk may lead to strategies to prevent complications in high-risk patients after ankle fracture fixation. With the goals of enhancing patient outcomes, lowering healthcare costs, and optimizing the use of healthcare resources, machine learning models may enable the identification of patients at high risk of SSI for tailored preventive treatments.

**Disclosures:**

**All Authors**: No reported disclosures

